# Late-onset Circulatory Collapse and Continuous Positive Airway Pressure are Useful Predictors of Treatment-requiring Retinopathy of Prematurity: A 9-year Retrospective Analysis

**DOI:** 10.1038/s41598-017-04269-5

**Published:** 2017-06-20

**Authors:** Mitsuru Arima, Shoko Tsukamoto, Kohta Fujiwara, Miwa Murayama, Kanako Fujikawa, Koh-Hei Sonoda

**Affiliations:** 1 Department of Ophthalmology, Kyushu University Graduate School of Medical Sciences, 3-1-1 Maidashi, Higashi-ku, Fukuoka City, Fukuoka 812-8582 Japan; 2 Department of Ophthalmology, National Kyushu Medical Center, 1-8-1, Jigyo-hama, Chuo-ku, Fukuoka City, Fukuoka 810-8563 Japan

## Abstract

Visual loss caused by retinopathy of prematurity (ROP) will be prevented if treatment-requiring ROP (TR-ROP) can be predicted. In this retrospective study including 418 infants with ≤32 weeks of gestational age (GA) and/or ≤1500 grams of birthweight, we attempted to identify useful predictors. We also examined the efficiency of significant predictors compared with existing predictive models, ROPScore and CHOP model. Multivariable logistic regression analyses supported the following factors were useful for predicting TR-ROP from all infants and infants with any ROP: GA (odds ratio [OR], 0.47 and 0.48), history of late-onset circulatory collapse (LCC) (OR, 2.76 and 2.44) and use of continuous positive airway pressure (CPAP) at 35 weeks of postmenstrual age (OR, 3.78 and 4.50). The comparison of areas under receiver operating characteristic curves indicated the combination of LCC, CPAP and ROPScore was better than ROPScore to predict TR-ROP from all infants and infants with any ROP (*P* = 0.007 and 0.02) and the combination of LCC, CPAP and CHOP model was also better than CHOP model to predict TR-ROP from all infants and infants with any ROP (*P* = 0.01 and 0.02). Our results suggested infants with a history of LCC and a long CPAP support have a high incidence of TR-ROP.

## Introduction

Retinopathy of prematurity (ROP) is a vasoproliferative disorder that occurs in the retina of preterm infants. Due to the improvement of the mortality rate of immature babies, the number of infants with ROP have tended to increase worldwide^1^. Although existing therapies such as laser photocoagulation, vitrectomy and intravitreal injection of antibody specific for vascular endothelial growth factor have been shown to have certain effects for the attenuation of ROP progression^2–4^, ROP is still a leading cause of visual loss in childhood. Because the provision of the optimal treatment at the proper time is essential to prevent ROP progression, the identification of more useful predictive factors for treatment-requiring ROP (TR-ROP) that can be used at earlier stages after birth is urgently needed.

Although the details of the pathogenesis of ROP have not been revealed, it is quite certain that retinal hypoxia is the core of ROP development. Retinal hypoxia is closely related to neovascularization and vascular hyper-permeability after the arrest of physiological retinal vascular growth, and the retinal hypoxia eventually enhances the formation of fibrovascular membrane which causes tractional retinal detachment^5, 6^. Oxygen transport to the tissue is disturbed by circulatory and pulmonary dysfunctions. Among such dysfunctions, (1) a decrease in blood pressure and effective circulating blood volume by an infection and circulatory disorder, (2) an insufficiency of blood oxygenation due to respiratory disease, and (3) a reduction in oxygen transport ability caused by anemia are representative conditions that induce tissue hypoxia. Our purpose in conducting the present study was to identify useful predictors for the development of TR-ROP from the standpoint of retinal hypoxia.

Specifically, we listed late-onset circulatory collapse (LCC), sepsis, patent ductus arteriosus (PDA) requiring ligation, anemia requiring blood transfusion, use history of oxygen, use of continuous positive airway pressure (CPAP) and bronchopulmonary dysplasia (BPD) as indicators of circulatory and pulmonary dysfunctions which could occur in newborns. We also listed intraventricular hemorrhage (IVH) and periventricular leukomalacia (PVL) as candidate factors which could cause retinal hypoxia, because they reflect the circulatory disorder in the central nervous systems (CNS). The relationship of these factors to the onset of TR-ROP was investigated in this study.

## Methods

This retrospective study was conducted under the approval of Institutional Review Board of Kyushu University Hospital and the principles of the Declaration of Helsinki. Informed consent regarding the use of data was obtained from each newborn’s parents.

### Patients

We analyzed the cases of neonates with ≤32 weeks of gestational age (GA) and/or ≤1500 grams of birthweight (BW) who were born at Kyushu University Hospital and underwent ophthalmic screening for ROP in a neonatal intensive care unit between November 2007 and November 2016. All infants were followed up until discharge at intervals indicated by ophthalmologists.

### ROP classification

The staging of the infants’ ROP was decided according to International Classification of Retinopathy of Prematurity Revisited^7^. We categorized all infants into three groups: TR-ROP, mild ROP and no ROP. Treatments were performed for infants with ‘type 1 ROP’ as described in the Early Treatment for Retinopathy of Prematurity Randomized Trial (stage 2 or 3 in zone II with plus disease, stage 3 in zone I with or without plus disease, or stage 1 or 2 disease in zone I with plus disease)^2^ or worse ROP, and these infants were classified into the group of TR-ROP. Infants with ROP of the less activity than TR-ROP were classified into the group of mild ROP. The stages of ROP adopted for statistical analyses were determined based on the stage and zone at the time of the infant’s initial treatment in the cases of TR-ROP, and at the time that the ROP had advanced the most in the cases of mild ROP. The day when we provided TR-ROP with the initial treatment or when mild ROP advanced most was defined as the day of ROP maturation.

### Predictive factors for TR-ROP

We investigated the following factors. GA, BW and sex were used as parameters of baseline at birth. As parameters of circulatory dysfunction, we examined LCC, sepsis, PDA requiring ligation, and blood transfusion before 35 weeks of postmenstrual age (PMA). LCC had been diagnosed by the review of medical records. Diagnostic criteria of LCC was summarized in Table 1. As parameters of pulmonary dysfunction, we examined the use history of oxygen, use of CPAP at 35 weeks of PMA, and BPD. The diagnosis of BPD was in accordance with described criteria^8^. We used IVH and PVL which occurred until 35 weeks of PMA as indicators of circulation in the CNS including the retina. We also examined the infants’ weight gain at 6 weeks after birth and the daily weight gain rate as parameters of growth after birth, which are used as parameters of existing predictive models, ROPScore and CHOP (Children’s Hospital of Philadelphia) model^9, 10^. The daily weight gain rate was calculated as described previously^11^. In brief, we divided the body weight change in one week from 8th to 15th postnatal day by 7 and defined this value as the daily weight gain rate in this study. We also confirmed whether significant predictors were beneficial to forecast TR-ROP by the comparison with ROPScore or CHOP model.

**Table 1:** Diagnostic criteria of LCC.

1. Several days have passed after the resolution of unstable circulatory condition in the acute postnatal phase.
2. Episodes of circulatory collapse as described below have appeared without obvious causes such as sepsis, PDA, hemorrhage, and other causes.
*Blood pressure*
Hypotension with ≤ 80% of blood pressure in the previous stable state
*Urinary volume*
(a) Oliguria with ≤ 50% of urinary volume in the previous stable state
(b) Oliguria with ≤ 1 ml/kg/hr over the past 8 hr
(c) Anuria for the past 4 hr (urinary retention is excluded)
3. Administration of glucocorticoid was required because treatment with volume expansion or a vasopressor was inadequate for the improvement of circulatory collapse.

### Statistical analyses

All analyses were carried out using SAS software (ver. 9.3, SAS, Cary NC). GA, BW, and weight gain at 6 weeks after birth were treated as continuous variables, and the other parameters were treated as categorical variables.

Because the current screening guideline for ROP uses only two factors of GA and BW which are recognized as universal risk factors for ROP^1, 6, 12, 13^, we calculated GA- and BW-adjusted odds ratios (ORs) and 95% confidence intervals (95% CIs). For predictive factors that were marginally significant (*P* < 0.1), we calculated the ORs and 95% CIs in multivariable-adjusted logistic regression analyses. To assess the ability of each predictive factor for TR-ROP over a range of values, we also calculated the area under the receiver operating characteristic (ROC) curve and compared the areas under the ROC curves (AUCs)^14, 15^. The cutoff values were obtained from the maximum values of Youden index (sensitivity + specificity − 1)^16^. A two-sided *P*-value < 0.05 was considered significant.

## Results

### Characteristics of infants in this study

We enrolled 418 infants in this study. Their characteristics are summarized in Table 2. Among them, 76 infants (18.2%) developed TR-ROP, 119 infants (28.5%) developed mild ROP, and 223 infants (53.3%) didn’t developed any ROP. The mean (standard deviation [SD]) PMA of ROP maturation in TR-ROP infants and mild ROP infants was 35.5 (2.3) weeks and 35.6 (2.7) weeks, respectively.

**Table 2:** Characteristics of the infants examined in this study.

	Total (n = 418)	TR-ROP (n = 76)	Mild ROP (n = 119)	No ROP (n = 223)	*P*-*value*
PMA of ROP maturation, mean (SD), week	—	35.5 (2.3)	35.6 (2.7)	—	0.68*
Predictive factors					
Baseline					
GA, mean (SD), week	28.8 (3.0)	24.8 (1.6)	27.7 (2.3)	30.8 (1.8)	<0.0001^†^
BW, mean (SD), g	1134 (453)	636 (206)	929 (291)	1414 (375)	<0.0001^†^
Male, No. (%)	221 (53)	44 (58)	63 (53)	115 (52)	0.75^‡^
Circulatory function					
LCC, No. (%)	95 (23)	50 (66)	34 (29)	11 (5)	<0.0001^‡^
Sepsis, No. (%)	36 (9)	20 (26)	9 (8)	7 (3)	<0.0001^‡^
PDA ligation, No. (%)	42 (10)	20 (26)	15 (13)	7 (3)	<0.0001^‡^
Transfusion, No. (%)	130 (31)	62 (82)	45 (38)	23 (10)	<0.0001^‡^
Pulmonary function					
Use history of oxygen, No. (%)	309 (74)	75 (99)	108 (91)	126 (57)	<0.0001^‡^
CPAP, No. (%)	138 (33)	56 (74)	39 (33)	43 (19)	<0.0001^‡^
BPD, No. (%)	120 (29)	60 (79)	41 (34)	19 (9)	<0.0001^‡^
Circulation in CNS					
IVH, No. (%)	62 (15)	25 (33)	26 (22)	11 (5)	<0.0001^‡^
PVL, No. (%)	23 (6)	8 (11)	8 (7)	7 (3)	0.048^‡^
Growth					
Weight gain at 6 weeks after birth, mean (SD), g	443 (278)	227 (137)	374 (195)	593 (292)	<0.0001^†^
Weight gain rate, mean (SD), g/day	10.5 (10.5)	3.1 (7.4)	6.5 (9.4)	15.0 (9.7)	<0.0001^†^

Abbreviations: ROP, retinopathy of prematurity; TR-ROP, treatment-requiring retinopathy of prematurity; PMA, postmenstrual age; GA, gestational age; SD, standard deviation; BW, birthweight; LCC, late-onset circulatory collapse; PDA, patent ductus arteriosus; CPAP, continuous positive airway pressure; PMA, postmenstrual age; BPD, bronchopulmonary dysplasia; CNS, central nervous systems; IVH, intraventricular hemorrhage; PVL, periventricular leukomalacia. *Unpaired Student’s *t*-test; ^†^Wilcoxon rank sum test; ^‡^chi-squared test.

### Prediction of TR-ROP from all infants

Table 3 shows the results of our GA- and BW-adjusted and multivariable-adjusted logistic regression analyses of candidate factors to predict TR-ROP among all 418 infants. The results revealed that GA (OR, 0.47; 95% CI, 0.35–0.61; *P* < 0.0001), a history of LCC (OR, 2.76; 95% CI, 1.24–6.19; *P* = 0.01), and the use of CPAP at 35 weeks’ PMA (OR, 3.78; 95% CI, 1.66–8.93; *P* = 0.002) were independent significant predictors.

**Table 3:** Predictive factors for TR-ROP in all infants.

Predictive factors	Crude		GA- and BW-adjusted*		Multivariable-adjusted	
	OR (95% CI)	*P*-*value*	OR (95% CI)	*P*-*value*	OR (95% CI)	*P*-*value*
GA	0.40 (0.33–0.49)	<0.0001	0.50 (0.40–0.64)	<0.0001	0.47 (0.35–0.61)	<0.0001
BW	0.994 (0.992–0.995)	<0.0001	0.997 (0.995–0.999)	0.003	0.999 (0.997–1.001)	0.23
Male	1.20 (0.73–1.99)	0.47	1.41 (0.67–3.02)	0.37		
LCC	12.69 (7.19–22.40)	<0.0001	3.12 (1.46–6.66)	0.003	2.76 (1.24–6.19)	0.01
Sepsis	7.28 (3.56–14.89)	<0.0001	1.89 (0.67–5.28)	0.23		
PDA ligation	5.20 (2.66–10.14)	<0.0001	1.54 (0.63–3.79)	0.35		
Transfusion	17.84 (9.43–33.77)	<0.0001	1.42 (0.57–3.56)	0.45		
Use history of oxygen	34.62 (4.75–252.25)	0.0005	2.12 (0.25–18.18)	0.49		
CPAP	8.88 (5.11–15.98)	<0.0001	3.94 (1.77–9.09)	0.007	3.78 (1.66–8.93)	0.002
BPD	17.63 (9.50–32.69)	<0.0001	1.35 (0.56–3.28)	0.50		
IVH	4.04 (2.25–7.27)	<0.0001	1.01 (0.43–2.38)	0.97		
PVL	2.57 (1.05–6.29)	0.04	3.91 (1.00–15.23)	0.05	2.96 (0.68–13.18)	0.15
Weight gain at 6 weeks after birth	0.995 (0.994–0.997)	<0.0001	0.999 (0.996–1.002)	0.56		
Weight gain rate	0.919 (0.892–0.944)	<0.0001	0.981 (0.937–1.030)	0.43		

Abbreviations: TR-ROP, treatment-requiring retinopathy of prematurity; GA, gestational age; BW, birthweight; OR, odds ratio; CI, confidence interval; LCC, late-onset circulatory collapse; PDA, patent ductus arteriosus; CPAP, continuous positive airway pressure; PMA, postmenstrual age; BPD, bronchopulmonary dysplasia; CNS, central nervous systems; IVH, intraventricular hemorrhage; PVL, periventricular leukomalacia. *GA is BW-adjusted and BW is GA-adjusted.

We next determined the ROC curves and calculated the AUCs to evaluate the utility of these factors (Supplementary Fig. 1, Table 4). The combination of GA and BW predicted TR-ROP among all infants with the AUC of 0.933 (95% CI, 0.904–0.963). When LCC and CPAP were added to GA and BW, the AUC became significantly larger (AUC, 0.950; 95% CI, 0.924–0.975; *P* = 0.006).

**Table 4:** AUCs for prediction of TR-ROP from all infants using combination of ROPScore, CHOP model and significant predictors.

	AUC	95% CI	*P-value*	*P-value*	*P-value*
GA + BW	0.933	0.904–0.963	Reference	—	—
ROPScore	0.935	0.906–0.963	0.57	Reference	—
CHOP model	0.935	0.906–0.965	0.21	—	Reference
GA + BW + LCC + CPAP	0.950	0.924–0.975	0.006	—	—
ROPScore + LCC + CPAP	0.951	0.926–0.976	0.005	0.007	—
CHOP model + LCC + CPAP	0.950	0.925–0.975	0.006	—	0.01

Abbreviations: AUC, area under receiver operating characteristic curve; ROP, retinopathy of prematurity; TR-ROP, treatment-requiring retinopathy of prematurity; CI, confidence interval; GA, gestational age; BW, birthweight; CHOP model, Children’s Hospital of Philadelphia model; LCC, late-onset circulatory collapse; CPAP, continuous positive airway pressure.

To confirm whether LCC and CPAP can be used to predict TR-ROP, we compared them with ROPScore and CHOP model, which are existing predictive models for ROP. ROPScore is based on GA, BW, weight gain at 6 weeks after birth, use history of oxygen, and blood transfusion^10^. CHOP model is based on GA, BW and weight gain rate^9^. We calculated the AUCs for ROPScore and CHOP model, and we found that they are not significantly larger than the AUC for the combination of GA and BW (*P* = 0.57 and 0.21, respectively). However, the combination of ROPScore, LCC and CPAP predicted TR-ROP more than both the combination of GA and BW and the ROPScore alone (AUC, 0.951; 95% CI, 0.926–0.976; *P* = 0.005 and 0.007, respectively). The combination of CHOP model, LCC and CPAP was also better than the combination of GA and BW and the CHOP model alone (AUC, 0.950; 95% CI, 0.925–0.975; *P* = 0.006 and 0.01, respectively). We additionally calculated the predicted probability of TR-ROP using the following equation: Probability of TR-ROP = 1/[1 + exp (−risk score)], where risk score = 18.738 + (−0.770) *GA + (−0.000824)*BW + 1.140*I (LCC) + 1.542*I (CPAP), where I (A) = 1 if A is present and 0 otherwise. Using the cutoff value of 0.252, the sensitivity was 92.9% and specificity was 89.1% for predicting TR-ROP from all infants.

### Prediction of TR-ROP among the infants with any degree of ROP

We conducted GA- and BW-adjusted and multivariable-adjusted logistic regression analyses of candidate factors for predicting TR-ROP among the infants with any degree of ROP (Table 5). These results also proved that GA (OR, 0.48; 95% CI, 0.36–0.64; *P* < 0.0001), a history of LCC (OR, 2.44; 95% CI, 1.08–5.56; *P* = 0.03), and the use of CPAP at 35 weeks of PMA (OR, 4.50; 95% CI, 1.91–11.09; *P* = 0.0005) were significant factors.

**Table 5:** Predictive factors for TR-ROP in infants with any degree of ROP.

Predictive factors	Crude		GA- and BW-adjusted*		Multivariable-adjusted	
	OR (95% CI)	*P*-*value*	OR (95% CI)	*P*-*value*	OR (95% CI)	*P*-*value*
GA	0.46 (0.37–0.58)	<0.0001	0.54 (0.42–0.69)	<0.0001	0.48 (0.36–0.64)	<0.0001
BW	0.995 (0.993–0.997)	<0.0001	0.998 (0.996–1.000)	0.03	1.000 (0.997–1.002)	0.81
Male	1.16 (0.62–2.07)	0.62	1.43 (0.67–3.08)	0.36		
LCC	4.81 (2.59–8.93)	<0.0001	2.70 (1.26–5.79)	0.01	2.44 (1.08–5.56)	0.03
Sepsis	4.37 (1.87–10.21)	0.0007	2.15 (0.74–6.26)	0.16		
PDA ligation	2.48 (1.18–5.21)	0.02	1.35 (0.55–3.32)	0.51		
Transfusion	7.28 (3.66–14.49)	<0.0001	1.45 (0.58–3.65)	0.43		
Use history of oxygen	7.64 (0.97–60.43)	0.05	1.57 (0.16–14.98)	0.70		
CPAP	5.74 (3.08–11.07)	<0.0001	4.60 (2.01–11.01)	0.0003	4.50 (1.91–11.09)	0.0005
BPD	7.13 (3.66–13.92)	<0.0001	1.53 (0.64–3.67)	0.34		
IVH	1.75 (0.92–3.35)	0.09	0.87 (0.37–2.02)	0.74		
PVL	1.63 (0.59–4.55)	0.35	3.70 (0.90–15.19)	0.07	2.80 (0.62–13.80)	0.18
Weight gain at 6 weeks after birth	0.994 (0.992–0.997)	<0.0001	0.999 (0.996–1.002)	0.48		
Weight gain rate	0.957 (0.922–0.990)	0.01	0.989 (0.944–1.039)	0.66		

Abbreviations: TR-ROP, treatment-requiring retinopathy of prematurity; GA, gestational age; BW, birthweight; OR, odds ratio; CI, confidence interval; LCC, late-onset circulatory collapse; PDA, patent ductus arteriosus; CPAP, continuous positive airway pressure; PMA, postmenstrual age; BPD, bronchopulmonary dysplasia; CNS, central nervous systems; IVH, intraventricular hemorrhage; PVL, periventricular leukomalacia. *GA is BW-adjusted and BW is GA-adjusted.

Similarly, we drew ROC curves and calculated the AUCs (Supplementary Fig. 2, Table 6). The AUC for the combination of GA and BW to predict TR-ROP among infants with any degree of ROP was 0.859 (95% CI, 0.803–0.916). When LCC and CPAP were added to GA and BW, the AUC became significantly larger (AUC, 0.898; 95% CI, 0.851–0.945; *P* = 0.009).

**Table 6:** AUCs for prediction of TR-ROP from infants with any degree of ROP using combination of ROPScore, CHOP model and significant predictors.

	AUC	95% CI	*P*-*value*	*P*-*value*	*P*-*value*
GA + BW	0.859	0.803–0.916	Reference	—	—
ROPScore	0.863	0.807–0.919	0.40	Reference	—
CHOP model	0.865	0.809–0.920	0.03	—	Reference
GA + BW + LCC + CPAP	0.898	0.851–0.945	0.009	—	—
ROPScore + LCC + CPAP	0.898	0.851–0.945	0.009	0.02	—
CHOP model + LCC + CPAP	0.900	0.854–0.946	0.007	—	0.02

Abbreviations: AUC, area under receiver operating characteristic curve; ROP, retinopathy of prematurity; TR-ROP, treatment-requiring retinopathy of prematurity; CI, confidence interval; GA, gestational age; BW, birthweight; CHOP model, Children’s Hospital of Philadelphia model; LCC, late-onset circulatory collapse; CPAP, continuous positive airway pressure.

To examine the efficiency of LCC and CPAP as predictors of TR-ROP, we compared them with ROPScore and CHOP model. Compared to the combination of GA and BW, the prediction of TR-ROP was not improved by the ROPScore (*P* = 0.40), but it was improved by the CHOP model (AUC, 0.865; 95% CI, 0.809–0.920; *P* = 0.03). The combination of ROPScore, LCC and CPAP predicted TR-ROP more than both the combination of GA and BW and the ROPScore alone (AUC, 0.898; 95% CI, 0.851–0.945; *P* = 0.009 and *P* = 0.02, respectively). The combination of CHOP model, LCC and CPAP was also better than the combination of GA and BW and the CHOP model alone (AUC, 0.900; 95% CI, 0.854–0.946; *P* = 0.007 and *P* = 0.02, respectively). We additionally calculated the predicted probability of TR-ROP using the following equation: Probability of TR-ROP = 1/[1 + exp (−risk score)], where risk score = 16.761 + (−0.718)*GA + 0.000159*BW + 1.088*I (LCC) + 1.701*I (CPAP), where I (A) = 1 if A is present and 0 otherwise. Using the cutoff value of 0.340, the sensitivity was 87.1% and specificity was 79.3% for predicting TR-ROP from infants with any degree of ROP.

## Discussion

To identify predictors of the development of TR-ROP from the standpoint of retinal hypoxia, we chose parameters related to circulatory and pulmonary dysfunctions and circulatory disorders in the CNS in addition to parameters of the immaturity of physique and development. The mean PMA that infants received laser photocoagulation was 35.2 weeks in the Early Treatment for Retinopathy of Prematurity Randomized Trial^17^ and the mean PMA of ROP maturation in infants with TR-ROP was 35.5 weeks. Therefore, we set the deadline for judging whether parameters of LCC, sepsis, PDA ligation, transfusion, use history of oxygen, CPAP, IVH and PVL were positive to 35 weeks of PMA. The feature of this study to be emphasized most is that a history of LCC was included as the candidate factor for predicting TR-ROP.

LCC is defined as a late-onset circulatory collapse that occurs suddenly in newborns whose circulation dynamics have stabilized once after leaving the acute phase after birth^18–20^. The circulatory failure in LCC does not respond to volume expansion or vasopressors, but it does respond to an administration of glucocorticoid^21^. Because LCC appears particularly in infants with early GA and low BW^19, 20^, it has been speculated that the incidence of LCC will increase.

Previous studies revealed that the responses of cortisol and adrenocorticotropic hormone (ACTH) to stimulation by corticotropin-releasing hormone were lower in premature infants than in full-term infants, and the basal value of cortisol was higher in preterm infants than in full-term infants^22, 23^. In other words, preterm infants may have a weakened ability to upregulate cortisol in response to various stresses although the basal secretion is preserved. The basal value of cortisol after birth begins to decline from the initial 1 week^22^, and LCC frequently appears at the same time. Because the circulation dynamics are markedly stabilized by an administration of glucocorticoid, it is thought that relative adrenal insufficiency is one of the major causes of LCC^19, 21, 24^. However, the endocrine function of premature infants remains unclear in many respects, and there is no definite opinion regarding the appropriate range for cortisol and ACTH concentrations in preterm neonates^25, 26^. For these reasons, LCC is defined only as a syndrome with ‘late-onset’ circulatory failure without obvious causes which responds to glucocorticoids at the present stage.

Although the etiology of LCC has not been clarified, it was demonstrated that the CNS blood flow is decreased in LCC patients^27, 28^, and that infants with LCC have a high incidence of IVH, PVL and pulmonary dysfunction^19, 20^. Several studies have revealed that LCC has a close relationship with circulatory and pulmonary failures which are also recognized as risk factors of ROP^6, 29, 30^, but our results of multivariable logistic regression analyses showed that LCC was independently related to the severity of ROP. The present study is the first to demonstrate that LCC is a useful predictive factor for TR-ROP.

As described in previous reports^19, 20^, LCC tended to occur in infants with shorter GA and lower BW (Supplementary Table 1). LCC appeared prior to ROP maturation in all infants with both LCC and TR-ROP. However, contrary to our expectations LCC did not give any significant difference in the characteristics of population with TR-ROP (Supplementary Table 2). The reason remains unknown, and the further investigation about the influence of LCC on retinal blood flow is urgently needed.

In the present investigation, we also examined the relationship of respiratory assistance with CPAP to the development of TR-ROP. The use of CPAP can achieve a reduction of ventilation- and oxygen-mediated lung injury by shortening the duration of intubation and the use of oxygen. Moreover, various systems of non-invasive CPAP have been developed such as nasal CPAP (n-CPAP) and biphasic n-CPAP, which are well-established modes of respiratory support for preterm neonates^31–34^. Although pulmonary dysfunction has certainly decreased with the spread of CPAP, conditions that require respiratory support still indicate an immaturity of pulmonary function. However, no consensus has been obtained regarding whether the use of CPAP is associated with the onset of TR-ROP. Our present analyses confirmed that a long CPAP support can be used to predict the development of TR-ROP. We speculate possibilities as follows; (1) CPAP contributes to the reduction of lung injury, but the injury does not completely disappear and (2) long-term support with CPAP implies that respiratory complications such as apnea remain, indicating inefficiency of blood oxygenation that ultimately promotes retinal hypoxia. Although there are both positive and negative opinions regarding the relationship between CPAP use and ROP development^35–38^, it is possible that this disagreement is caused by difference in the characteristics of the studies’ patients. The current screening guideline for ROP is based on GA and BW which are recognized as universal risk factors for ROP worldwide^1, 6, 12, 13^. We therefore adjusted all parameters with GA and BW before conducting the statistical analyses, and the results revealed that long-term support with CPAP is a significant predictor of TR-ROP. However, a prospective and large-scale trial is needed before making any firm conclusions, of course.

Our findings also indicate that a history of LCC and a long CPAP support can be used to help predict TR-ROP, based on the AUC results. We observed that the model combining LCC, CPAP, GA and BW was significantly better than the model combining GA and BW for the prediction of TR-ROP both among all infants and among infants with any degree of ROP. We also calculated AUCs using the ROPScore and the CHOP model from among the various predictive models for ROP because their parameters could be extracted from our data base^11, 39^. The prediction of TR-ROP from all infants was not improved by using the ROPScore or CHOP model alone compared to the model using GA and BW. The prediction of TR-ROP from infants with any ROP was not improved by using the ROPScore, but it was improved by using CHOP model compared to the model using GA and BW. On the other hand, the prediction of TR-ROP both from all infants and from infants with any ROP was significantly improved by using the model combined with GA, BW, LCC and CPAP. The prediction was markedly improved by the addition of LCC and CPAP use to each model compared with the model using GA and BW. Surprisingly, either model combining LCC, CPAP and the ROPScore or CHOP model was better than the ROPScore or CHOP model alone. These results supported that LCC and CPAP have possibilities to become valuable indicators. However, we originally should update the value of weight gain rate and re-calculate the risk score on a weekly basis when using CHOP model. But in this study we divided the weight change from 8th to 15th postnatal day by 7 and used this value as weight gain rate as described previously^11^. This is the limitation of this investigation. To confirm whether this new predictive model combined with GA, BW, LCC and CPAP is better than existing clinical models, the prospective and multicenter study is required.

Another important point to be highlighted is that both LCC and CPAP use are easy to use as parameters. The current screening guideline recommends that the initial eye examination should be performed within 31 weeks of PMA or within 4 weeks after birth^12^. Considering that the mean (SD) PMA and postnatal day of LCC onset in our patient series were 29.3 (2.5) weeks and 22.3 (12.7) days (Supplementary Table 1), it is possible that we can obtain the crucial information before the initial examination. We can also determine at a glance whether CPAP support is continued or not. Because no complicated procedure is required, we speculate that ROP prediction using the two parameters of LCC and CPAP use will be beneficial for busy clinical ophthalmologists.

We identified useful predictive factors for TR-ROP. Our findings suggest that preterm infants with a history of LCC and long-term respiratory support by CPAP are at high risk of developing TR-ROP.

## Electronic supplementary material

Supplementary information
