# Supplementary material for: Late-onset Circulatory Collapse and Continuous Positive Airway Pressure are Useful Predictors of Treatment-requiring Retinopathy of Prematurity: A 9-year Retrospective Analysis
Source: Sci Rep. 2017 Jun 20;7:3904. doi: 10.1038/s41598-017-04269-5 (PMC5478650; doi:10.1038/s41598-017-04269-5)
Supplement: Supplementary file 1 — Supplementary information [file 41598_2017_4269_MOESM1_ESM.pdf]

Supplementary information for

Late-onset Circulatory Collapse and Continuous Positive Airway Pressure Are Useful Predictors of Treatment-requiring Retinopathy of Prematurity: A 9-year Retrospective Analysis

Mitsuru Arima, Shoko Tsukamoto, Kohta Fujiwara,

Miwa Murayama, Kanako Fujikawa and Koh-Hei Sonoda

Supplementary figure 1. ROC curve for the prediction of TR-ROP among all 418 infants

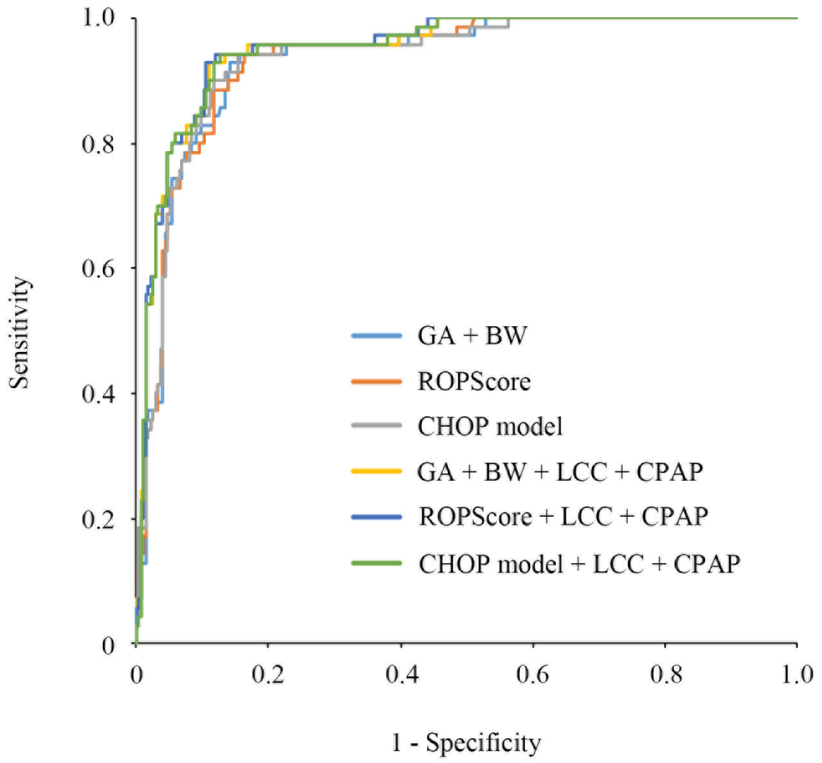

Abbreviations: ROC, receiver operating characteristic; ROP, retinopathy of prematurity; TR-ROP, treatment-requiring retinopathy of prematurity; GA, gestational age; BW, birthweight; CHOP model, Children's Hospital of Philadelphia model; LCC, late-onset circulatory collapse; CPAP, continuous positive airway pressure

Supplementary figure 2. ROC curve for the prediction of TR-ROP among the infants with any degree of ROP

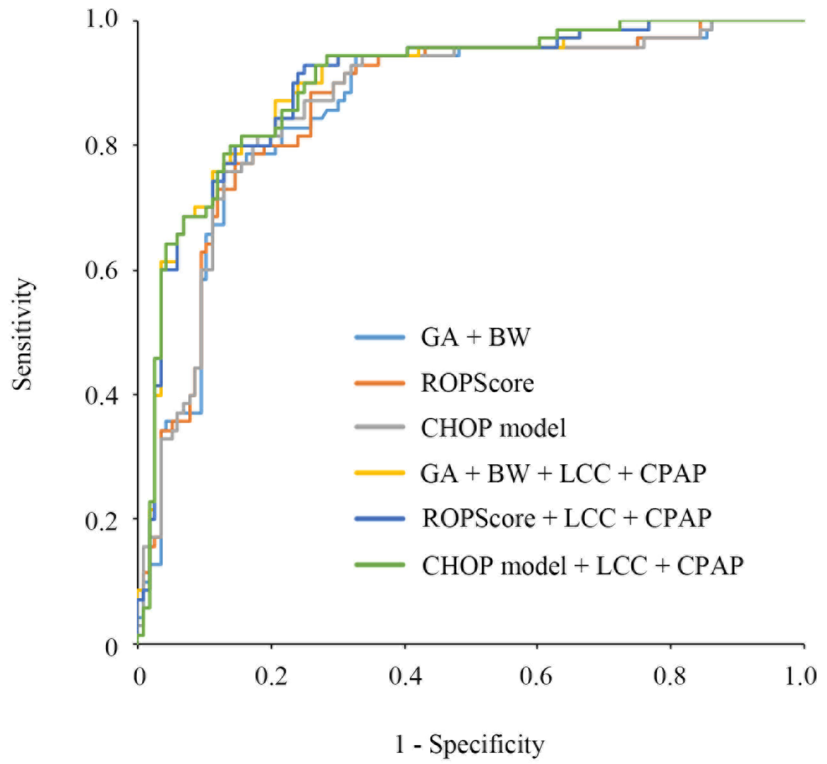

Abbreviations: ROC, receiver operating characteristic; ROP, retinopathy of prematurity; TR-ROP, treatment-requiring retinopathy of prematurity; GA, gestational age; BW, birthweight; CHOP model, Children's Hospital of Philadelphia model; LCC, late-onset circulatory collapse; CPAP, continuous positive airway pressure

Supplementary table 1. Characteristics of infants with LCC among all infants

	**Total (n=418)**	**LCC (n=95)**	**No LCC (n=323)**	***P-value****
PMA of LCC onset , mean (SD), week	–	29.3 (2.5)	–	–
Postnatal day of LCC onset , mean (SD), day	–	22.3 (12.7)	–	–
Baseline				
GA, mean (SD), week	28.8 (3.0)	26.1 (2.5)	29.6 (2.7)	<0.0001
BW, mean (SD), g	1134 (453)	769 (350)	1242 (423)	<0.0001

Abbreviations: LCC, late-onset circulatory collapse; PMA, postmenstrual age; SD, standard deviation; GA, gestational age; BW, birthweight. * = unpaired Student's *t*-test

Supplementary table 2. Characteristics of infants with LCC among infants with TR-ROP

	**Total (n=76)**	**LCC (n=50)**	**No LCC (n=26)**	***P-value****
PMA of LCC onset<br>, mean (SD), week	–	28.3 (2.1)	–	–
Postnatal day of LCC onset<br>, mean (SD), day	–	24.8 (14.7)	–	–
PMA of ROP maturation<br>, mean (SD), week	35.5 (2.3)	35.6 (2.3)	35.2 (2.2)	0.46
Postnatal day of ROP maturation<br>, mean (SD), day	74.6 (16.8)	76.0 (15.3)	72.0 (19.3)	0.33
Baseline				
GA, mean (SD), week	24.8 (1.6)	24.7 (1.6)	25.0 (1.8)	0.45
BW, mean (SD), g	636 (206)	624 (202)	660 (217)	0.47

Abbreviations: LCC, late-onset circulatory collapse; ROP, retinopathy of prematurity; TR-ROP, treatment-requiring retinopathy of prematurity; PMA, postmenstrual age; SD, standard deviation; GA, gestational age; BW, birthweight. * = unpaired Student's *t*-test
